# Neurobrucellosis Complicated by Sensorineural Hearing Loss: A Case Report

**DOI:** 10.7759/cureus.29482

**Published:** 2022-09-23

**Authors:** Bayan Mirza, Hala M Kanawi, Talal Alkhatib, Afnan F Bukhari, Faisal Zawawi

**Affiliations:** 1 Department of Medicine and Surgery, College of Medicine, Umm Al Qura University, Makkah, SAU; 2 Otolaryngology–Head and Neck Surgery, King Abdulaziz University, Jeddah, SAU

**Keywords:** sensorineural hearing loss, infection, audiogram, sensorineural deafness, neurobrucellosis

## Abstract

Brucellosis is a zoonotic disease. It is also one of the neglected infectious diseases and is less well-known compared to other diseases. It is acquired from infected animals (cattle, sheep, goats, camels, pigs, or other animals) through the consumption of unpasteurized dairy products or contact with tissues or fluids. Sensory neural hearing loss (SNHL) in neurobrucellosis had been described in the literature, mostly as an incidental finding that otolaryngologists should consider in any patient with fever and a history of travel to the Middle East, Central or South America, or other brucellosis-endemic countries. We present a neurobrucellosis case with profound bilateral SNHL that was treated with combination antibiotic therapy for long periods of time and highlight the clinical course of the patient.

## Introduction

Brucellosis is one of the most widespread zoonotic infections worldwide [1]. It is an endemic disease in Saudi Arabia [2]. Although the disease is found worldwide, the Mediterranean region, Middle East, Africa, and Latin America are the main areas of public health concern [2]. A study found that brucellosis annually infects over 500,000 people in sub-Saharan Africa [2]. It is caused by Brucella, a gram-negative, non-motile, non-spore-forming coccobacillus and a facultative intracellular organism [3]. There are approximately 12 species of Brucella, and the most common species causing brucellosis worldwide are Brucella melitensis (from sheep and goats), Brucella abortus (from cows), and Brucella canis (from dogs) [2].

Most infections are transmitted to the human population through direct contact with fluids from infected animals and consumption of their food products, including unpasteurized milk and cheese [3]. In addition, occupational or recreational exposure to infected animals and their products, such as in farmers or veterinarians, can increase the risk of brucellosis [1]. There is also evidence of transmission from mother to child via breastfeeding [4].

Brucellosis is a multisystemic infection that affects any organ in the body. Neurobrucellosis (NB) is the most severe yet uncommon complication of brucellosis. It can involve the central and peripheral nervous systems, with a prevalence of 1.7% to 10% of brucellosis worldwide [5]. This study aimed to describe a case of NB with a history of progressive bilateral hearing loss.

## Case presentation

This case was reviewed by biomedical ethics, and an exemption was received. A 35-year-old man who did not have any medical illness was referred to our institution complaining of rapidly progressing bilateral hearing loss for 18 months. It is associated with recurrent episodes of fever, sweating, fatigue, and walking difficulty. He also experienced headaches, back pain, and joint pain. He had no history of otorrhea, tinnitus, vertigo, weight loss, reduced appetite, or history of exposure to ototoxic medications or noise trauma. He had a history of raw cheese and milk consumption in Yemen three years ago for approximately two years. On examination, the patient was conscious and alert but was severely affected by bilateral hearing loss. His body weight was 50 kg, height was 160 cm, and vital signs were within normal limits.

**Figure 1:**
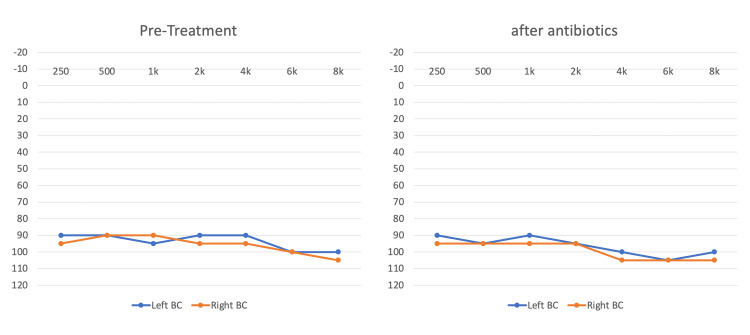
Audiogram pre and post-antibiotic therapy This figure highlights the audiometer testing performed prior to therapy and after the conclusion of therapy showing bilateral profound sensorineural hearing loss indicating a lack of improvement in both ears.

On neurological examination, the power in the lower limbs was graded 4/5 with a normal tone. Symmetrically brisk deep tendon reflexes in the upper and lower limbs with a positive Babinski sign on the right side. Sensory examination results were normal. Gait was normal. A cranial nerve examination revealed bilateral sensory neural deafness. All other cranial nerves were normal. The meningeal signs were negative. His cardiovascular and respiratory systems were unremarkable. Pure tone audiometry revealed profound bilateral sensorineural hearing loss (SNHL) with bilateral type A tympanograms (Figure 1).

**Figure 2:**
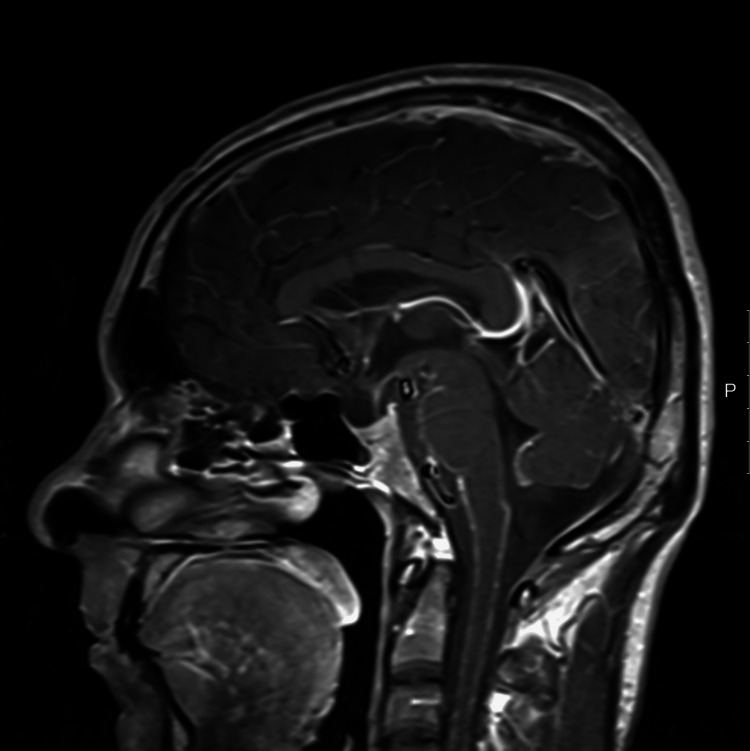
MRI brain scan with contrast showing perimesencephalic and cervical spinal cord leptomeningeal enhancement, as well as cranial nerve enhancement

Magnetic resonance imaging (MRI) was performed, and perimesencephalic and cervical spinal cord leptomeningeal enhancement, as well as cranial nerve enhancement, was seen (Figure 2).

The brucellosis screening test revealed positivity for Brucella antibody with a titer of 340 (normal range < 80) and blood examination of hemoglobin was 13.5 gm/dl, white cell count was 3,500/mm with normal differential count, and C-reactive protein (CRP) level of 60 mg/L (normal range 0-3 mg/L). Renal and liver function tests were within normal limits. Cerebrospinal fluid (CSF) analysis showed CSF WBC (white blood cell count) was 560 cells/cubic, RBC (red blood cells count) 18 cells/cubic, proteins 7.25 g/l, sugar 1.0 mmol/L (normal range 2.3-4.0). Brucella species was isolated in CSF. No other organisms were detected in the CSF Gram or acid-fast stains.

The patient was admitted to the hospital as a case of neurobrucellosis (NB) based on lumbar puncture (LP) analysis. The medical and infectious disease teams treated the patient with intravenous doxycycline, rifampicin, and ceftriaxone for six weeks. After six weeks, his symptoms, including fever, night sweats, fatigue, and walking difficulty, improved but his repeated audiogram remained unchanged. After nine months, multiple audiograms and an auditory brainstem response (ABR) test showed persistent profound bilateral SNHL (Figure 1). The patient was then referred to the cochlear implant program for cochlear implantation.

## Discussion

Brucellosis is considered a major health issue in countries in the Middle East, including Saudi Arabia. It is the most common zoonotic infection worldwide [2]. However, the disease is highly neglected by the World Health Organization (WHO) and World Organization for Animal Health (OIE) [6,7]. It is endemic to Saudi Arabia, with an infection rate of approximately 70 per 100,000 people [2,8]. We reviewed several studies conducted in various regions of Saudi Arabia. Human brucellosis is considered one of the most commonly reported cases, especially in Riyadh [1]. In the southern region of Saudi Arabia, a study was conducted among 4,900 patients; 2.3% had active disease and 19.2% had serological evidence of brucella antigen exposure [1]. While in a study of 1,733 patients conducted in Riyadh, the prevalence of brucellosis was 8.8% [1]. The principal causative factors in this region are unpasteurized raw milk intake and interaction with contaminated animals due to the common practice of drinking raw milk mainly from sheep and camels [9,10].

There is no consensus on the exact diagnosis of NB because it has neither a conventional clinical picture nor clear CSF findings [11,12]. Therefore, the diagnostic criteria suggested in the literature [5,13,14] are as follows:

1) The presence of consistent clinical symptoms, either with meningitis or meningoencephalitis

2) Consistency of typical CSF findings with meningitis (protein concentrations > 50 mg/dL, leukocytes > 10/mm3 and glucose to serum glucose ratios < 0.5)

3) Positive bacterial culture or serological test results for brucellosis in blood specimens (positive Rose Bengal test and serum tube agglutination with a titer ≥ 1/160), CSF (positive Rose Bengal test or serum tube agglutination with any titer), or positive bone marrow culture

 4) Findings in cranial computed tomography (CT) or magnetic resonance imaging (MRI)

Brucellosis has a variable clinical appearance owing to its broad systemic involvement during contamination. Involvement of the central nervous system (CNS) is an uncommon but severe complication of the disease course. It may often be the only sign of human brucellosis, with a prevalence of 1.7% to 10% worldwide [5]. Meningitis, which can be acute or chronic, is the most common presentation of NB, accounting for 17% to 74% of all cases. Other neurological manifestations include encephalitis, meningoencephalitis, radiculitis, myelitis, peripheral and cranial neuropathies, subarachnoid hemorrhage, psychiatric manifestations, brain abscesses, and demyelinating syndrome [12,15].

Cranial nerve (CN) involvement is common in brucellosis, particularly in the sixth, seventh, and eighth CNs. The acoustic nerve was identified as the cranial nerve most frequently involved [16,17]. When Brucella toxin enters the labyrinth, it affects the cochlear system, causing damage and hearing loss [18]. According to Thoma et al., there is no adequate literature on NB cases with sensorineural hearing loss. As a result, otolaryngologists were unaware of this [18]. In NB, SNHL is usually bilateral and mainly affects high frequencies, and our patient had profound bilateral SNHL affecting all frequencies.

In NB, rifampicin, doxycycline, and trimethoprim/sulfamethoxazole are effective for long periods. These chemotherapeutics penetrate the central nervous system and exert synergistic effects. Ceftriaxone can be added as a fourth agent to this protocol [19,20]. Our patient received doxycycline, rifampicin, and ceftriaxone for six weeks.

Brucellosis-induced CN paralysis usually improves without residual effects following antibiotic administration [21]. Chronic CNS infections, on the other hand, often result in permanent neurologic deficits. In our patient, after six weeks of antibiotics, the audiograms remained unchanged and inconsistent with the case report of Thomas et al. [18], which presented a one-year history of bilateral SNHL at a high frequency and received tetracycline and rifampicin for a total of six weeks with no improvement in hearing. On the other hand, CN paralysis completely improved after treatment, according to Ucmak et al. [22].

There are only sporadic reports of SNHL as a complication of NB. One of the early reports was by Bucher et al. [23], who diagnosed SNHL in a patient with severe chronic NB. Thereafter, Thomas et al. [18], Bedur et al. [24], Cagatay et al. [25], and Sengoz et al. [26] all reported similar outcomes in patients with NB developing SNHL. Interestingly, all reported cases (including our case) had suffered ataxia and gate-related issues in addition to SNHL, indicating cerebellar involvement [18,23-26].

Therefore, prevention is an essential aspect of disease control, and vector management is crucial when dealing with zoonotic infections. Pasteurizing milk and handling raw meat safely are essential preventive measures. In addition, animals should be examined and vaccinated regularly. Finally, an educational program for healthcare workers and veterinary and cattle owners should be considered [1,2].

## Conclusions

Otolaryngologists should not overlook NB as a cause of hearing loss. It should be investigated in any patient presenting with hearing loss, especially when other differential diagnoses have been ruled out or if habitual or demographic history puts the patient at a higher risk of acquiring NB.
